# Cyberbullying in the Digital Age: Prevalence and Associated Factors

**DOI:** 10.1192/j.eurpsy.2025.1138

**Published:** 2025-08-26

**Authors:** A. Guedria, N. Maatallah, R. Ayoub, A. Riahi, K. Faidi

**Affiliations:** 1 Child and Adolescent Psychiatry Departement, Fattouma Bourguiba Hospital; 2 University of Monastir, Monastir, Tunisia

## Abstract

**Introduction:**

Cyberbullying has emerged as a significant concern in today’s digital age, particularly among adolescents. As technology becomes increasingly integrated into daily life, the prevalence of online harassment has risen, impacting the mental health and well-being of students.

**Objectives:**

This study aims to explore the prevalence of cyberbullying among Tunisian schoolchildren and to identify the associated factors that contribute to its occurrence.

**Methods:**

This is a cross-sectional descriptive and correlational study among students from two public middle schools in the Sousse governorate (Tunisia) for the year 2020/2021 using an information sheet concerning: Socio-demographic data, schooling, peer relationships, child’s use of the Internet and also the ‘Cyberbullying Screening Test’ which is a self-administered questionnaire designed to assess cyber-bullying behaviour among young people aged 12 to 18.

**Results:**

We included 238 middle-school students.63.6% of the population were girls, with a sex ratio of 0.57. The majority of participants (83.1%) was between 13 and 15 years old. More than half of middle school students (51.3%) reported having been cyberbullied at least once and 30.2% having been sexually harassed at least once by cell phone or on the Internet. We identified a significant association between cyberstalking and the mother’s level of education (P=0.041). There was a statistically significant relationship between cyberbullying and the student’s place of residence (P=0.023). School repetition among students was also associated with cyberbullying with a strong significance (P=0.002). A notable relationship was found between cyberstalking and the frequency of digital technology use on weeends (P=0.055).

**Conclusions:**

The findings of this study reveal a significant prevalence of cyberbullying among Tunisian middle school students, with notable associations linked to many environmental and individual factors. These results emphasize the urgent need for comprehensive strategies to combat cyberbullying and support affected students in educational environments.

**Disclosure of Interest:**

None Declared

